# Dental Hints Department

**Published:** 1907-10

**Authors:** B. H. Teague

**Affiliations:** Aiken, S. C.


### ...DENTAL HINTS DEPARTMENT...

CONDUCTED BY B. H. TEAGUE, D. D. S., AIKEN, S. C., TO WHOM ALL CONTRIBUTIONS TO THIS DEPARTMENT SHOULD BE SENT

When wheels become caked from grinding amalgam use with them a paste of carborundum flour in glycerine.

When investing a large bridge the investment may be strengthened by placing a ring of iron wire round the bridge, but taking care that it does not touch it.—Dr. H. L. Dowell, Record.

MUST SHOW PHOTO TO GET LICENSE.—Every applicant for a license to practice dentistry in Pennsylvania hereafter will be required to present with his application for examination by the State Dental Board a photograph of himself certified by the dean of the college from which he was graduated, and signed by the candidate himself.—Dental Digest.

Full bridges have a tendency to contract, so that the two ends represented by the molar crowns are drawn towards one another, this may be overcome by inserting a piece of the stem of a clay pipe in the investment, adjusting it so that the ends fit tight against the two crowns, it may also advantageously be used on any bridge which is much curved. —Dr. H. L. Dowell, Record.

THYMOCAMPHENE.—As a disinfectant canal dressing, to two drams each of thymol and phenol one dram of camphor gum is added. Mixed in a dry test tube, and fused with low heat, the camphor thoroughly dissolves, and the result is a stable liquid at ordinary temperatures.—Dental Register.

CEMENT IN COMBINATION WITH ABSORBENT COTTON AS A SEALING DRESSING.—A happy method which I have followed for some years, where cement is used to seal medicaments in teeth under treatment, more especially when devitalizing pulps, is to incorporate in a thin mix sufficient absorbent cotton to produce a mass slightly less in size than the cavity. This is easily placed without pressure on the pulp, and more easily removed than when cement alone is used. Try it.—Joseph Loran Pease, Pacific Dental Gazette.

Oxy Chloride of Zinc for Lining Cavities.—Since oxy phosphate of zinc becomes saturated with septic germs when used as a filling material, it is better to use oxy chloride of zinc for lining cavities and filling in under large fillings of metal.

Preparation of Amalgam for a Filling.—To make a strong filling, and one that will have close margins, get first the right proportion of mercury. Mix and work the material until the mass is smooth and plastic. It should then take the finest skin markings of the fingers. Continue the kneading until the first slight evidence of stiffening becomes apparent, and then make the filling quickly. It should stiffen at once on being pressed into the cavity, so that it will not draw back from the walls when the instrument is withdrawn, or move again when more material is pressed upon it.—G. V. Black, Dental Review.

Preservation of Silex.—To prevent detoriation of the silex, never put a brush that has been used on the cast into it. The exceedingly small amount of sulphate of lime that will adhere to the brush will cause milkiness and spoiling of the material—rather pour out a small quantity onto the cast and spread with a swab of cotton. Also, if thickened, dilute with water that has been boiled to free it from lime.—Dr. J. H. Beebee, Dental Practice.

To Perfume Vaseline.—Much of the vaseline sold today has an unpleasant odor and this is especially noticeable when it is used for lubricating disks and strips in finishing fillings. The odor may be overcome by adding to the vaseline a few drops of one of the essential oils—whichever one the operator may prefer. This does not in any way impair the vaseline and it makes it more agreeable to use.—C.N.Johnson,Review.

ARTIFICIAL DENTURES.—As with natural dentures, the lips should be capable of free and easy movement over the teeth and the labial surfaces of the artificial dentures. The margins of the plate and its external contours should not encroach upon the line of action of any of the muscles concerned in these movements. The labial contours of the plate and the teeth should support the lips in those positions which have been deemed proper in the establishment of the fixed expıession of the face.—Charles R. Turner, Philadelphia, Pa., Review.

TO MAKE CEMENT ADHERE TO ENAMEL.—Sometimes when the surface of enamel near the gum becomes exceedingly sensitive, the surest way of relieving it is to flow some cement over it and let it remain till it wears off. To make the cement stick to the smooth surface of the enamel, smear some of the liquid of the cement over the sensitive area and let it remain a few minutes before adapting the cement. The material will then not flake off.—J.

Dr. Clements of Macon, Miss., years ago showed by microscopic work that the cementum of the teeth affected with pyorrhea alveolaris was more dense than that of sound teeth, and that this was due to a deposition of calcium salts in the canaliculi, the salts displacing the organic tissues therein and thus destroying the vital union of the tooth with the surrounding tissues. Dr. Smith also speaks of the density of structure of teeth afflicted with pyorrhea alveolaris.—Dr. J. V. Baswell, Digest.

COIN GOLD.—The best gold that I can find—that which lasts the longest and looks the best—for it keeps its form and lustre and withstands in a greater degree the stress of mastication, is the American coin. It is also cheaper. A $10 gold piece costs $10, and contains 232.2 grains of pure gold which, if made into 22 K. and purchased of the dealer, would cost $11.08. The alloy of the American gold is fixed by law, to be of copper and silver, the silver not to exceed 10 per cent. of the copper. This gold, being 900 fine, is, therefore, 21.6 K. fine.—Dr. J. H. Beebee, Dental Practice.

To Re-Bake an Inlay.—If for any reason you desire to rebake a porcelain inlay after you have removed the platinum matrix, make an investment of powdered soap stone and thin shellac varnish, while investment is fresh imbed the inlay cavity side down until flush with margins. Dry slowly, and you may fuse as high as you like with no change to margins or shape. This would probably not do for low fusing porcelain, because the shellac would probably not all burn out and a discoloration would develop. The soap stone powder may be made by scraping a fresh mechanic's crayon.—J. M. Evey, Dentist's Magazine.

Porcelain Homogeneous.—Porcelain in its entirety is a homogeneous mass, with the maximum strength that naturally belongs to it, but it is reduced the moment a foreign material is added, and while porcelain has a greater affinity for platinum than any other metal, it does not lessen the fact that it is still a foreign matter; therefore, knowing this, it is recommended that pins and loops are only used in cases where it is unavoidable, and that percentage is probably one-fifth or less.—Dr. W. A. Capon, Dominion Dental Journal.

Cement a Tooth Saver.—Now it is a recognized fact that gold can be so inserted in teeth of good structure with healthy surroundings that it will stand the test of years, while in a poor grade of teeth where bacteria and their effects run riot, it is very difficult, if not impossible, to preserve them with gold alone. Under such conditions we fill these teeth with cement, feeling sure there would be no decay under the filling, or we would use an inlay, with the assurance of the inlay workers, that it would preserve the tooth, where a gold filling would last but a short time, showing our reliance upon cement as a tooth-saver.—Dr. E. A. Royce, Dominion Dental Journal.

Blood Supply and Disease.—That the question of blood supply enters into the production of the disease is shown by the fact that pyorrhea alveolaris rarely attacks a pulpless tooth and its progress upon an affected tooth is retarded by the removal of the pulp. An affected tooth is nearly always benefited by the removal of its pulp, and although it has never been definitely stated why this is the case, it is most probably due to the change in the blood supply in the cementum and pericementum, the withdrawal from the dentin probably allowing an increase of nutrition to those structures, hence an increased vitality.—Dr. J. V. Boswell, Digest.

Treatment for Gum Before Setting Crown.—When the gum has been wcunded in grinding the root or in reducing the band to root level, the acid of the cement works into the wound and gives rise to discomfort similar to that following the application of salt or vinegar. This, of course, subsides as the cement sets, but is anything but pleasant while it lasts. To overcome the pain thus inadvertently induced and to head off such as may attend the setting of the crown, I dry the root and adjoining gum and paint them with chloropercha. The effect is immediate and the discomfort hardly worthy of mention.—J. C. Eddington, Dental Office and Laboratory.

Suggestive Anesthesia.—The use of hypnotism in tha dental profession was given a successful test, April 20th, in the Dental Department of the Medico-Chirurgical College of Philadelphia. The test consisted in pulling a tooth that had an abcess at the root and had been broken off at the crown. No pain whatever was felt and the only consciousness the patient had of anything going on was that he felt the tooth slipping from his jaw. The one to introduce this science into the college was William E. Hoffman, a junior student in the department. Hypnotism as a means of making dentistry painless has been tried by several institutions and dentists with more or less success, and, it is claimed, has been experimented with in the Medica-Chirurgical Hospital for the past two years.—Dental Digest.

Misconceptions Concerning Porcelain: Don't Jar the Body.—It is fatal to the best results and the greatest density of porcelain to jar the body into place, as is usually done.

When a mass of wet porcelain is jarred it causes air bubbles to settle toward the bottom of the mass, and the more it is jarred the larger are the bubbles. This may be proved by placing porcelain on the end of a spatula and jarring it. Then examine it with a magnifying glass, and it will be found full of bubbles near the bottom of the mass. Porcelain should be gently pressed or wiped into place with a spatula, in such a manner as to constitute a burnishing of the material. When built up in this way it is very dense and strong, and there is little shrinkage in fusing it. I seldom find it necessary to fuse a jacket-crown more than once in any tooth, anterior to a molar. I use the highest-fusing body I can get, and have no need for more than four shades. —Louis Ladewich, Dental Review.

Cement Under Gutta-Percha.—The use of cement under gutta-percha has given me greater satisfaction than any other application of this method of filling. To stop a tooth with gutta-percha is usually regarded as a very simple operation, but if a really good filling is to be made the material must be placed with much care. The tenderness which occurs under gutta-percha fillings tells us they are far from moisture proof. Soft cement placed in the cavity and the gutta-percha forced into it will give a filling that absolutely stops the cavity—It will have the non-conducting qualities of the gutta-percha and the cavity sealng quality of cement.—Dr. A. E. Royce, Review.

Office Assistant.—Were I asked what I regarded as the most indispensable item of my equipment, I should unhesitatingly reply, "My office assistant." A practice must be very small indeed that cannot afford the magic touch of a woman's hand. A mere girl who has just left the public school can soon be trained to do a multitude of things that consume time, and still do them as well as the dentist; and if they are in the nature of housekeeping she will do them better. Only the dentist who employs one, and has had to without her for a day or two, can know her value.—Dr. H. Clark, Dominion Dental Journal.

Cement Lining.—Dr. Cochran, of Burlington, Iowa, has used cement lining under gold fillings for six years in a manner that is very satisfactory to him. A matrix of forty-five gauge pure gold is formed to fit the cavity, and after adjusting the rubber, Ames' slow setting cement is placed in the cavity and the matrix forced to place. Then the gold filling is inserted, using a band matrix if indicated. Dr. Cochran uses Pack's pellets or non-cohesive gold, finishing with heavy foil. I have found that some form of crystal gold works to good advantage in many cases.—Dr. A. E. Royce, Review.

Cover for Cement.—If you will stop to think for one moment what you are doing when you make an inlay, you will realize that you are simply putting cement into a cavity with a protection or cover which may be of gold, porcelain or whatever you like. If that be the case, it is only a question in my mind as to which is the best cover for that cement, and I am free to confess that I have yet to see very few cases outside of bridge anchorages, even including approximal cavities in molars and bicuspids, where I can not put in porcelain that will do just as good service as any gold inlay.—Dr. F. C. Van Woert, Items.

Cement Under Gold Fillings.—W. Thompson Madin, D. D. S., of Birmingham, England, gives a method of using cement under gold fillings that seems to solve the problem very nicely, and it places the gold filling ahead of the inlay as a tooth saver according to the ideas advanced by the inlay advocates. His method is to prepare the cavity as for a gold filling and cover the walls with cement as for an inlay. Into this he forces thirty (30) gold foil as if for a matrix for an inlay. The filling is then inserted into this matrix in the usual manner. This gives a practical and expeditious method of using cement under gold fillings.—Dr. A. E. Royce, Review.

Sulphuric Acid Disintegrates.—There was a time when we dwelt heavily on the use of sulphuric acid in the treatment of putrescent pulps. Treat such a tooth thoroughly with sulphuric acid, and in from six months to two years, the patient may come back with the tooth crown broken. That occurs many times, but few of us realize the cause. The fact of the matter is that sulphuric acid disintegrates the teeth and leaves no life in them. I only use sulphuric acid in the treatment of putrescent pulp when I expect to crown them.—Dr. Oakman, Register.

Extracting Teeth for Nothing.—There is no more absurd practice among the many unbusinesslike acts of a dentist than that of extracting teeth for nothing. That a busy dentist will devote the time necessary to removing all the useless teeth from the jaw, with all the unpleasant, even dangerous, associations connected therewith, without fee or reward, is almost incomprehensible. Many a second or third-rate surgeon receives a fee of fifty dollars or more for an operation occupying much less time and skill, but the dentist performs this for the privilege of, six months hence, making a denture for ten dollars, and probably waiting six months more for his pay. This act looks like a bonus for having teeth removed and replaced by artificial substitutes. —Dr. J. H. Irwin, Dominion Dental Journal.

Color of Each Tooth.—As a general rule the upper centrals and laterals are nearly or quite the same color, the upper canines are yellower, and the remaining teeth a compromise between the two, and possibly darker. The lower teeth are nearly or about the same color, which is of rather a lighter shade than the upper canines. Color, we know, depends greatly on light, its direction, intensity, etc. It also depends on the translucency of the object under consideration. Two teeth may have exactly the same amount of coloring with the light from all directions, but they will differ markedly when placed in front of some dark object.—Alex. Jameson, Dental Review.

Gold Fillings in Porcelain.—To put a gold filling in a mineral tooth, grind an impression in the tooth where the filling is required, then cover the ground surface with a littel

Jenkins' inlay body, and on this press a piece of sponge gold the same size. Fuse this in furnace, and then condense gently with engine mallet, add gold and finish in the same way as with a gold filling. If required, a tooth may be entirely faced with gold in this manner, giving the appearance of an all gold crown, the inlay body holding the first layer of gold quite firmly, if ordinary care is taken as with a gold filling.—Chas. Every Brown, British Dental Journal.

Destroy Pulps in Pyorrhoea.—In these cases, the pulps of all teeth should be removed; do not try to compromise with the trouble by removing the pulps only of the very loose teeth, for the following reasons: Firstly, if you do such operations, the teeth that are still firm will in all probability become loose if their pulps are allowed to remain, and secondly, the unaffected teeth are needed to support those already in trouble, and again, if you remove the pulps only of the loose teeth and attach them to support each other, you have to a large extent burnt the bridge behind you and rendered far more difficult a complete operation, which will be indispensable if the dentition is to be permanently preserved. —Dr. E. Eggleston, Summary.

Manipulating Moldable Porcelain.—I believe that the model method is preferable for adjusting the material. There are many accessible cavities that can be kept dry for a reasonable length of time, and it can be done much more rapidly by molding directly to the cavity. In the class of cases where it is indicated, however, I think it is desirable to make a model. You merely prepare the cavity, take an impression and dismiss the patient. If your model is not perfect, the final fitting may be advantageously made in the mouth. The fitting is done directly to the cavity, and the questionable feature of the accuracy of the model is eliminated.—F. E. Roach, Chicago, Review.

Practice Economy.—A cry is often raised that dentistry is not a lucrative calling, and this is true in the sense that great wealth cannot be accumulated in its practice. And yet these matters are all relative and a large fortune is not needed by a professional man. Every dentist, if he will discipline himself to economy and care, can save something each year for the proverbial rainy day. Saving is so much a matter of habit that I am often impelled to beg of young men entering the profession to take careful stock of their possibilities in this direction and to make one resolve at the very beginning of their careers. This is, that each year they will spend less than they make. In doing this it is seldom that they will be deprived of any essential element of comfortable livelihood.—Dr. C. N. Johnson, Review.

Injection of Hydrogen Peroxide.—We should not inject into a small fistula hydrogen peroxide full strength, as the effervescence may result in trauematic infection. Many cases of carious bone or necrosis have resulted from injections into blind abscess pockets. The fistula may be at the end of the root of the tooth, but when you inject peroxide into the tooth canal it is not always possible to get it to pass out freely through the external orifice of the fistula. The sooner dentists stop the use of peroxide of hydrogen in root canals, and more especially in those of the lower jaw, the less caries and necrosis there will be to deal with.—Dr. Oakman, Register.

Absorption of Deciduous Roots.—I think it has been conceded that the roots of the deciduous teeth do not absorb physiologically after the death of the pulp, and in that belief I have been in the habit of retaining deciduous teeth in place, and yet allowing eruption of the permanent teeth by grinding away the entire crown of the deciduous molar, in that way allowing its expulsion from the alveolus by the oncoming permanent tooth. I sometimes would leave the pulp canals entirely open, instructing the mother as to the care of these. With single-rooted teeth, the time comes when these should be removed, whether the roots have absorbed or not as the ends of these roots are sometimes diverted sideways and without extraction there is likely to be trouble.—W. V. B. Ames, Chicago.

Combination of Gutta-Percha and Cement.—Personally, I have found the mounting of crows with gutta-percha very difficult, and recently have been using a combination of gutta-percha and cement for many cases. The gutta-percha is placed upon, or in, the crown, as the case may be, and while soft the crown is pressed to place, and after removal of crown and gutta-percha the root is smeared with the soft cement and the crown replaced, allowing the cement to cover the root and cement the gutta-percha to it. In the use of Asher's cement it is very desirable to line the cavity with a good inlay cement. The adhesive qualities of the latter seems to keep the Asher's cement in its place and to make a better adaptation to the cavity walls.—Dr. A. E. Royce, Review.

Soft Cohesive Gold.—Owing to the exact knowledge and especial skill required to work and adapt surely to cavity walls, this stiff cohesive gold is accountable for many poor fillings. From the same "batch" of gold prepared as non-cohesive (designated then as "soft"), the softness was due to the lack of cohesion—the sliding of particles by each other instead of clinging to each other at every point of contact, and not to any difference in the purity of the gold. That is to say, non-cohesive gold can be produced which is soft in the nature of it, and cohesive gold can be produced that is soft in working qualities—in folding on itself until the pressure for condensing is put upon it, becoming rigid at the desired moment and not materially so before.—R. B. Tueder, American Dental Journal.

Prophylaxis.—The deep thinkers of the medical profession admit that if they can bring their prophylaxis to such a degree of perfection as to eliminate disease they will have accomplished their highest mission. If we can follow the same course in the practice of dentistry, we shall be the highest idealists of our time, and that is the ***somnum bonum*** of life. The profession and the public alike must be educated before these higher ideals can be grasped and practised. This is a hard matter to accomplish because of the apathy which exists in the world, but it is certain to come. It is difficult to break off from old habits. It is well to know that mere mechanics is not the highest attainment so far as dentistry is concerned. —Alfred Owro, Review.

Earning Years.—The best earning years of a dentist do not extend over twenty years, and during these years he should endeavor to arrange to save from fifteen to twenty thousand dollars, otherwise he runs a great risk of being compelled to stint himself and his family in later years. We should not allow ourselves to be misguided by the sight of dentists who live to a ripe old age and remain able to earn a good livelihood right to the last. These men are the exception, not the rule, and usually possess constitutions much above the average. The fact that it is not possible for a dentist to acquire great wealth from the practice of his profession should discourage no one. It is certainly not the wealthy who really enjoy life or are really to be envied. We must all observe this fact sooner or later. The man who really enjoys life is he who has to work for a moderate income and who lives within his means, satisfying himself with the gradual but sure method of accumulating a modest competence.—Dr. W. C. Tratter, Dominion Dental Journal.

Impression for Gold Inlay.—Let us suppose that we have a proximal cavity in a bicuspid, and a molar is adjoining it. I take an impression by bending a piece of metal at right angles, and in that I place the dental manufacturing modeling compound, because it is a low heat material. I press it to place and get an overflow. I then take it out of the cavity, chill with ice water, trim away the overflow, leaving one thirty-second of an inch that is not in contact with the margin, and place it into the cavity again. It will go in like an inlay. I now place a wedge in proximally, and, with pressure on the grinding surface, drop warm water on very slowly until I see it is yielding under the pressure, and then reset it. After chilling it and taking it out I have an impression which is as accurate as an inlay should be with no element of uncertainty as to whether the margins have been distorted or not.—W. H. Taggart, Chicago, in Review.

Calcific Degeneration.—It has been my privilege on two occasions to examine teeth that were apparently of as sound nature as teeth could be, and the individuals so far as their histories were concerned had never been troubled with any dental interference. The individuals died from arterial sclerosis; the teeth were examined, along with a post-mortem examination, and it was found that the pulps in the thirty-two teeth had all undergone calcific degeneration. Calcific infiltration or a tendency to such conditions in an individual have a fundamental bearing upon the dental organs, and it has been my observation of several years of close study that a person having the long bluish-white teeth have a predisposition for the formation of lime salts in certain tissue structure. In a vast majority of these cases it will also be observed that the skin has rather a bluish-pink color, and which shows that the venous circulation is in some way interfered with. If occasion should demand the extraction of teeth in such individuals it would be well to observe whether or not the pulps of the teeth have undergone calcific degeneration. I think a close observation will reveal such a fact.—Dr. Geo. W. Cook, American Dental Journal.

Treating an Abcess.—Take modeling composition and get it soft enough to take an impression of the sore tooth and one or two teeth on each side. Let the impression come well up on the buccal side. Have it thick enough to hollow out in shape to get one of Colson's Dental Poultices (made by Dr. C. B. Colson of Charleston, S. C.) in place next to the abcess. Deepen the bite of the sore tooth. This application will serve three purposes. It will hold poultice in place, protect the cheek and rest the afflicted tooth. It will only require five minutes extra time and will be a great comfort to the patient who would have such a tooth to nurse for twenty-four to forty-eight hours or longer.—Dr. J. W. Gray, Darlington, S. C.

Drug Remedies.—Since putrefactive changes in the intestines and non-destruction of waste products are largely due to hepatic insufficiency, the liver now requires attention. A

remedy that will stimulate the liver and cause a flow of bile may be selected from a half dozen or more preparations. A few will be named in the order of their excellence. The dentist should try all these drugs and methods upon himself to note the results. Calomel may be given by itself or in combination with podophyllin, soda, ipecac, etc. Given alone it should be prescribed in 1-12 to 1-6 grain every hour until one grain is taken. This is to be followed with a saline laxative. Podophllin (mayapple, mandrake) has good results, but is slow in acting though a direct and positive hepatic stimulant. It acts upon the glandular system of the alimentary canal. In small repeated doses it produces ptyalism. As an hepatic stimulant, it requires four to eight hours for action which may last from one to two days. This drug should be taken in small doses, to prevent griping. One-twelfth to one-sixth grain hourly, until one-half grain is taken. A little sodium chloride will aid its action and will not leave constipation in its wake. Podophyllin should never be given in large doses. Calomel and podophyllin may be combined with excellent results. Dr. Abbott recommends the following:

Calomel......................1-6 grain.
Podophyllin.................1-67 grain.
Rhein.........................1-6 grain.
Capsicum .................1-134 grain.

Together, hourly, from 6 to 10 p. m. A saline laxative is used next morning before breakfast. I have used the following prescription with good results:

Aloin..........................1-4 grain.
Strychnine Sulphate......1-6 grain.
Extract Belladonna ......1-8 grain.
Py. Ipecac.................1-16 grain.

Taken at bedtime. In prescribing either of these, a good saline cathartic should be taken the next morning upon rising to flush the intestinal tract and remove the accumulation that has caused the decomposition. Flushing the bowels with a saline laxative at the proper time to cleanse out the intestinal tract is imperatively indicated. If this be not accomplished when digestion and absorption is re-established excretory material will again be taken up by the portal system and the condition desired will not be obtained.—Dr. E. S. Talbot, Dentists' Magazine.

